# MicroRNA159 Can Act as a Switch or Tuning MicroRNA Independently of Its Abundance in Arabidopsis

**DOI:** 10.1371/journal.pone.0034751

**Published:** 2012-04-12

**Authors:** Maria M. Alonso-Peral, Cheng Sun, Anthony A. Millar

**Affiliations:** 1 Research School of Biology, Australian National University, Canberra, Australian Capital Territory, Australia; 2 Plant Industry, Commonwealth Scientific and Industrial Research Organisation, Canberra, Australian Capital Territory, Australia; Rutgers University, United States of America

## Abstract

The efficacy of gene silencing by plant microRNAs (miRNAs) is generally assumed to be predominantly determined by their abundance. In Arabidopsis the highly abundant miRNA, miR159, acts as a molecular “switch” in vegetative tissues completely silencing the expression of two *GAMYB-like* genes, *MYB33* and *MYB65*. Here, we show that miR159 has a diminished silencing efficacy in the seed. Using reporter gene constructs, we determined that *MIR159* and *MYB33* are co-transcribed in the aleurone and embryo of germinating seeds. However in contrast to vegetative tissues, *MYB33* is not completely silenced. Instead, miR159 appears to shape the spatio-temporal expression pattern of MYB33 during seed germination. Transcript profiling in a time course during seed germination in wild-type and a *mir159* mutant in which miR159 is almost absent, revealed that transcript levels of the *GAMYB-like* genes were similar between these two genotypes during germination, but much higher in the *mir159* mutant once germination had completed. This attenuation in the silencing of the *GAMYB-like* genes was not explained by a decrease in mature miR159 levels, which remained constant at all time points during seed germination. We propose that miR159 acts as a tuner of *GAMYB-like* levels in Arabidopsis germinating seeds and that the activity of this miRNA is attenuated in the seed compared to vegetative tissues. This implies that the efficacy of miRNA-mediated silencing is not solely determined by miRNA abundance and target transcript levels, but is being determined through additional mechanisms.

## Introduction

MicroRNAs (miRNA) are ∼21 nt long RNA molecules that post-transcriptionally regulate the expression of target genes. By binding to the transcripts to which they are complementary, they trigger either mRNA destabilization or inhibit their translation [1], [2], [3]. When a miRNA and a target gene are co-transcribed in the same cell, the miRNA may completely silence the expression of the gene, in which case it is regarded as a “switch” miRNA. Alternatively if the level of target gene expression is reduced but not abolished, it is considered a fine “tuning” miRNA [4]. To our knowledge, it is unknown what defines these two different kinds of action, but presumably miRNA and target transcript abundance, and the complementarity between the two, would be factors influencing the silencing outcome. Considering the biological importance of miRNAs in regulating gene expression, it is not surprising that miRNAs are themselves under tight regulation [5]. miRNA transcription, biogenesis, degradation and function can be regulated by different mechanisms to modify the final activity of a miRNA [4].

Our work focuses on the study of the miRNA family miR159 of *Arabidopsis thaliana*. This family contains three members, miR159a-c, with miR159a and miR159b being the most abundant forms [6], [7]. These two miRNAs are functionally redundant, as single T-DNA insertional *mir159a* or *mir159b* mutants are morphologically indistinguishable from wild-type, but a *mir159ab* double mutant showed widespread developmental defects [8]. This miRNA pair is functionally specific for genes encoding two GAMYB-like transcription factors, *MYB33* and *MYB65*. Despite the existence of at least another 15 putative target genes containing the miR159 target site in their mRNA [7], [8], only *MYB33* and *MYB65* were de-regulated in *mir159ab* and all the developmental abnormalities of this double mutant were rescued in a *mir159ab.myb33.myb65* quadruple mutant [8].

Many lines of evidence suggested that miR159 acts as a molecular switch in vegetative tissues, completely silencing the expression of *MYB33* and *MYB65*. Firstly, in a study utilising the reporter lines *miR159-GUS*, *MYB33-GUS* (*MYB33* promoter and genomic regions fused to the β-glucuronidase gene) and *mMYB33-GUS* (identical to *MYB33-GUS* but carrying synonymous mutations in the miR159 target site rendering it resistant to miR159 regulation) demonstrated that miR159 and *MYB33* are co-transcribed in the same cells, but that MYB33 protein appears completely absent from vegetative tissues [8], [9]. Secondly, from genetic and transgenic approaches, we estimated that less than 10% of wild-type miR159 levels are enough to completely silence *MYB33* and *MYB65* in vegetative tissues, based on phenotypic and molecular analyses [7], [8]. Finally, *myb33.myb65* mutant plants display a wild-type phenotype at the vegetative stage and micro-array analyses performed on 15 day-old shoot apices found that the transcriptome of *myb33.myb65* was indistinguishable from wild-type [9]. All these observations imply that MYB33 and MYB65 protein levels are reduced to biologically insignificant levels in wild-type vegetative tissues by miR159 [9].

MYB33-GUS protein is present only in anthers and seeds where *MYB33* and *MYB65* are involved in the progression of Programmed Cell Death (PCD) of the aleurone in the seed, and the tapetum in the anther [9], [10]. However, previous studies have determined that miR159 is present in Arabidopsis seeds during germination [11]. Reyes and Chua (2007) detected high levels of miR159 in the seed by Northern blotting, and the *miR159a-GUS* and *miR159b-GUS* reporter genes are strongly expressed in embryos of imbibed seeds [8]. In addition to MYB33-GUS activity in seeds [10], functional analysis has demonstrated that three *GAMYB-like* genes are important in the progression of aleurone PCD, as a triple *myb33.myb65.myb101* mutant displayed altered aleurone vacuolation [9] implying the presence of these proteins in the seed.

In this paper we aimed to explain the apparent paradox of the coexistence of miR159 and the GAMYB-like proteins in the Arabidopsis germinating seed, as it has been previously shown that the co-transcription of *MIR159* and its targets results in the complete silencing of the latter in vegetative tissues. We have determined that despite being co-transcribed in the same tissues in the seed, MYB33 protein is still present and this is not explained by a decrease in miR159 levels. We propose that the activity of miR159 is attenuated in the seed compared to vegetative tissues, implying that the silencing outcome of miRNA-mediated regulation involves mechanisms in addition to miRNA abundance and complementarity to target genes.

## Results

### Comparison of miR159 and *MYB33* mRNA levels in germinating seeds and vegetative tissues

**Figure 1:**
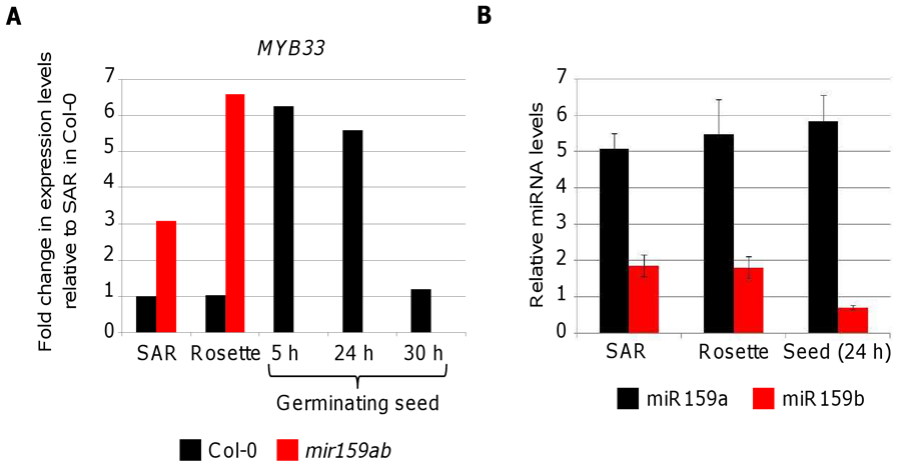
*MYB33* mRNA and mature miR159 levels are high in germinating seeds. (A) Relative *MYB33* expression levels were measured by quantitative RT-PCR in Shoot Apical Regions (SAR) of 15-day-old plants, 32-day-old rosettes and germinating seeds 5, 24 and 30 h after imbibition, and normalized to *CYCLOPHYLIN5*. Levels in wild-type SAR were then set to one and the rest of measurements were normalized to this tissue. Black bars correspond to Col-0 and red bars to *mir159ab* tissues. (B) Mature miR159a (black bars) and miR159b (red bars) levels in the same SAR, rosettes and 24 h-germinating seeds of Col-0 as measured by TaqMan microRNA assays. MicroRNA levels are relative to the small RNA *sno101*. Error bars represent standard deviation.

To gain insight into the regulatory relationship between *MYB33* and miR159 in seeds, we determined the levels of *MYB33* mRNA and miR159 in germinating seeds and compared them to vegetative tissues. We measured by qRT-PCR the steady-state mRNA levels of *MYB33* in different tissues of Col-0; shoot apical regions (SAR), rosettes and germinating seeds 5, 24 and 30 h after imbibition (Fig. 1A). We found that *MYB33* mRNA levels in 5 and 24 h germinating seeds were almost six-fold higher compared to SAR, rosettes and 30 h imbibed seeds. We also measured the *MYB33* mRNA levels in SAR and rosettes of *mir159ab* and found that these were similar to the levels in 5 h and 24 h germinating Col-0 seeds (Fig. 1A). This result suggested that *MYB33* mRNA levels are not regulated by miR159 early during germination. One possible explanation would be that *MIR159* is not expressed in 5 and 24 h germinating seeds. To test this hypothesis, we decided to measure mature miR159a and miR159b levels in SAR, rosettes and 24 h germinating seeds of Col-0. Surprisingly, we found that the levels of miR159a were the same in all three tissues and that the levels of miR159b were only 2 fold lower in seeds (Fig. 1B). There could be a number of reasons explaining the high levels of both *MYB33* transcript and miR159 in germinating seeds. For instance, *MYB33* and *MIR159* could be transcribed in different tissues within the seed, and therefore miR159 would be spatially unable to regulate *MYB33*. Alternatively, *MYB33* and *MIR159* may share the same transcriptional domain, but miR159 activity could be attenuated in seeds. We decided to carry out experiments to discern between these two scenarios.

### *MIR159* and *MYB33* are co-transcribed in aleurone and embryo during seed germination, where miR159 tunes *MYB33* expression

**Figure 2:**
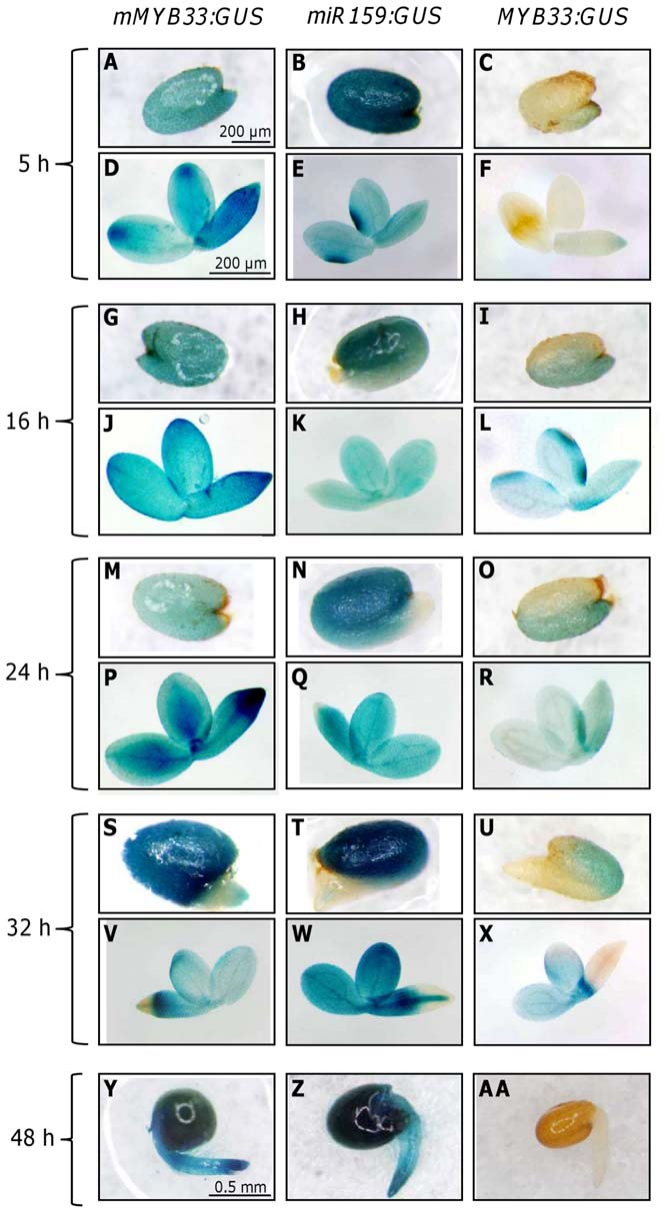
Expression pattern of *mMYB33-GUS*, *miR159b-GUS* and *MYB33-GUS* during seed germination. Expression pattern of *mMYB33-GUS* (A, D, G, J, M, P, S, V, Y), *miR159b-GUS* (B, E, H, K, N, Q, T, W, Z) and *MYB33-GUS* (C, F, I, L, O, R, U, X, AB) in the aleurone (A, B, C, G, H, I, M, N, O, S, T, U, Y, Z, AB) and in the embryo (D, E, F, J, K, L, P, Q, R, V, W, X, Y, Z, AB) of germinating seeds 5, 16, 24, 32 and 48 h after imbibition. Seed coat was removed to uncover the aleurone and seeds are oriented so the radicle is at the bottom of the picture. Scale bar for all the aleurones and all the embryos is the same as in A and D respectively, except for Y, Z and AB in which the scale bars is the same as in Y.

To determine if miR159 can potentially regulate the *GAMYB-like* genes during seed germination we studied the expression pattern of the reporter lines *miR159b-GUS*, *mMYB33-GUS* and *MYB33-GUS* at different time points during seed germination (Fig. 2). *MiR159a-GUS* and *miR159b-GUS* lines had been previously found to have a very similar expression pattern [8] and we found the same situation during seed germination, but only *miR159b-GUS* pictures are shown as this reporter gave stronger activity. A stronger activity from the *miR159b-GUS* reporter may appear counterintuitive as mature miR159a levels are much higher than miR159b (Fig. 1B), but previously qRT-PCR analysis had found pri-*MIR159b* levels much higher than pri-*MIR159a* which may indicate stronger promoter activity [8]. However as *pri-MIRNA* steady state levels will be determined by both transcript production (promoter strength) and transcript decay (*pri-MIRNA* processing efficiency), it could be speculated that a poor processing efficiency of pri-*MIR159b* is a strong contributing factor to this discrepancy between primary and mature miRNA levels.

**Figure 3:**
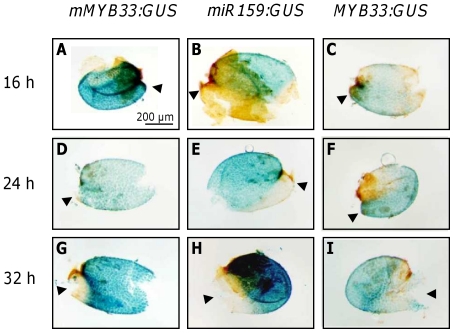
Isolated aleurone layers of *mMYB33-GUS*, *miR159b-GUS* and *MYB33-GUS* during seed germination. Dissected aleurone layers of stained *mMYB33-GUS* (A, D, G), *miR159b-GUS* (B, E, H) and *MYB33-GUS* (C, F, I) seeds. Seeds were stratified and kept in the growth chamber for 16, 24 and 32 h prior to staining. Arrowheads point to the part of the aleurone in contact with the radicle tip.

For the experiment we stained seeds 5, 16, 24, 32 and 48 h after imbibition. After 24 h the seed coat had cracked but the radicle had not penetrated the aleurone (Fig. 2 M, N, O). After 32 h the embryo radicle had penetrated the aleurone and protruded (Fig. 2 S, T, U) and after 48 h the radicle had elongated and exhibited root hairs (Fig. 2 Y, Z, AA). We analysed three different lines for each construct, and found that their staining pattern was indistinguishable. The construct *mMYB33-GUS* was highly expressed in the aleurone and embryo at all the time points tested (Fig. 2 A, D, G, J, M, P, S, V, Y; Fig. 3 A, D, G). The staining pattern was largely homogeneous with the only exception of 32 h germinating seeds in which staining was weaker in the embryo radicle tip and the corresponding area in the aleurone (Fig. 2 S and V; Fig. 3G). This ubiquitously strong staining suggests that *MYB33* is highly transcribed in the aleurone and embryo during seed germination. Germinating seeds of the line *miR159b-GUS* also stained very strongly at all time points tested (Fig. 2 B, E, H, K, N, Q, T, W, Z). In seeds imbibed for 5 h, staining was homogeneous and strong in both aleurone and embryo, but at the time points 16, 24 and 32 h the staining was weaker in the area of the aleurone that is in contact with the radicle (Fig. 2 H, N, T; Fig. 3 B, E, H), and for the embryo the radicle tip was also weakly stained after 24 and 32 h (Fig. 2 K, Q, W). In conclusion, *MIR159b* appears to be also strongly transcribed, in a domain that largely overlaps with *MYB33* transcription.

To examine where MYB33 protein is expressed, the *MYB33-GUS* reporter lines were stained. Surprisingly, unlike what happens in rosette tissues, MYB33-GUS protein was detectable in germinating seeds in tissues where *MIR159* is also transcribed (Fig. 2 C, F, I, L, O, R, U, X, AA). In 5 h germinating seeds, very weak GUS staining was detected only in the radicle tip (Fig. 2 F) and in the part of the aleurone which is in contact with the radicle tip (Fig. 2 C). However, after 16 and 24 h of imbibition, the whole embryo appeared weakly stained. Staining also appeared to occur in the majority of aleurone cells, but was stronger in the region in contact with the radicle (Fig. 2 I, L, O, R; Fig. 3 C, F). This aleurone staining pattern was opposite and complementary to the one seen in the *miR159b-GUS* aleurone, suggesting that miR159 is spatially regulating MYB33 expression in this tissue. In 32 h *MYB33-GUS* germinating seeds, all aleurone cells were stained but staining was stronger in the area of the aleurone that is in contact with the cotyledons (Figs. 2 U and 3 I). In embryos, cotyledons and hypocotyl stained blue but the GUS signal was absent in the radicle, in a pattern identical to the *mMYB33:GUS* transgene (Fig. 2 X). Once germination had completed (48 h after imbibition), *mMYB33-GUS* and *miR159b-GUS* were found to be strongly expressed in the seedlings (Fig. 2 Y, Z), however *MYB33-GUS* expression was completely absent (Fig. 2 AA). This time course suggests that unlike vegetative tissues where miR159 is acting as a molecular switch completely repressing *MYB33-GUS* expression, miR159 may be acting as a tuner of MYB33 expression in germinating seeds.

### miR159 activity is attenuated in germinating seeds

**Figure 4:**
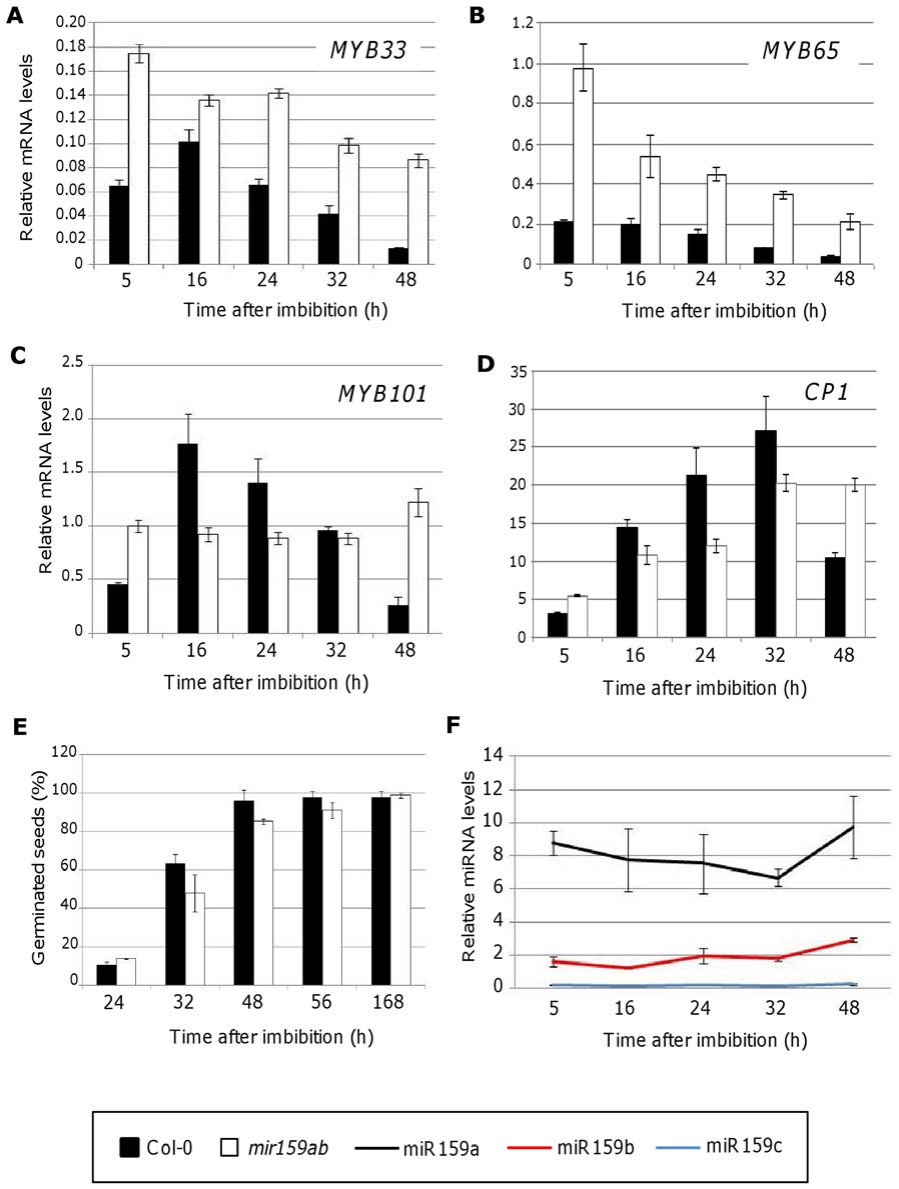
Transcript and miRNA profiling during seed germination. Relative mRNA levels of *MYB33* (A), *MYB65* (B), *MYB101* (C) and *CP1* (D) in germinating Col-0 (black bars) and *mir159ab* (white bars) seeds. Measurements were carried out by quantitative RT-PCR and were normalized to *CYCLOPHYLIN5*. (E) Germination rate of Col-0 (black bars) and *mir159ab* (white bars) seeds. (F) Mature miR159 levels in Col-0 germinating seeds as measured by Taqman microRNA assays. MiRNA levels are relative to the small RNA *sno101*. Error bars represent standard deviations.

To ascertain the extent of regulation of miR159 over *MYB33*, we decided to measure the steady-state levels of the *GAMYB-like* genes by qRT-PCR on wild-type and *mir159ab* germinating seeds (Fig. 4 A). We reasoned that if miR159 was regulating *MYB33* in germinating wild-type seeds, then *MYB33* mRNA levels should be higher in *mir159ab* compared to wild-type. We also measured the mRNA levels of *MYB65* and *MYB101*, two other *GAMYB-like* genes present in the seed (Fig. 4 B, C). Seeds imbibed for 5 h had *MYB33* mRNA levels that were almost 3 times higher in *mir159ab* seeds compared to wild-type. However, this difference was diminished 16 h after imbibition, as *MYB33* levels were similar in both backgrounds. In seeds imbibed for 24 and 32 h, the difference in *MYB33* levels between wild-type and *mir159ab* increased again, being 2.06 and 2.35 times higher in the *mir159ab* mutant seeds. In 48 h *mir159ab* germinating seeds, *MYB33* mRNA levels were 6 times higher than in wild-type, the same difference that has been previously described for rosettes [8, this work]. These results suggest that miR159 regulation of *MYB33* is diminished 16, 24 and 32 h after imbibition compared to the 5 h and especially 48 h time points. Similar trends were observed for *MYB65* and *MYB101* (Fig. 4B, C). Compared to wild-type, *mir159ab MYB65* mRNA levels were approximately 5-fold higher in seeds imbibed for 5 h. This fold-level differences then falls to 2.5–3 fold difference at 16 and 24 h, then increases to 4 then 5.5 fold in 32 and 48 h imbibed seeds respectively. Interestingly, in the case of *MYB101*, the levels of mRNA were even higher in the wild-type seeds 16 and 24 after imbibition (Fig. 4C).

Consistent with these changes in *GAMYB-like* mRNA levels are the changes in mRNA levels of a downstream gene, *CYSTEINE PROTEINASE1* (*CP1*), whose mRNA levels have been previously shown to reflect GAMYB-like protein activity [9]. *CP1* mRNA levels in wild-type seeds gradually increase during imbibition, but then decrease considerably at 48 h. This follows the pattern of mRNA levels of *MYB33*, *MYB65* and *MYB101*, but with a lag in the time at which the mRNA levels peak, probably reflecting that GAMYB-like protein synthesis is an additional step between the profiles. Conversely, *CP1* levels in *mir159ab* gradually increase but do not decrease at the 48 h time-point, reflecting that miR159 no longer represses the *GAMYB-like* genes at this time. The relative flat increase in *CP1* levels of *mir159ab* plants, compared to the “pulse” of *CP1* levels in wild-type, illustrates the requirement of miR159 to produce the dynamic changes in *CP1* during germination. Finally, the germination rate of wild-type and *miR159ab* seeds was scored at different time points for seven days and found to be similar between these two backgrounds (Fig. 4E) which supports the notion that all the changes in gene expression found in *mir159ab* seeds are due to the absence of the miRNA and not to an impaired germination process.

One possibility that would explain the attenuation of miR159 regulation upon *GAMYB-like* genes during germination is that despite being highly transcribed, primary *MIR159* transcripts are not being efficiently processed and therefore mature miR159 levels are low in germinating seeds 16, 24 and 32 h after imbibition. To determine if this is the case, we measured mature miR159 levels by TaqMan MicroRNA Assays (Fig. 4F). Interestingly, miR159 levels in wild-type germinating seeds were as high as in vegetative tissues at all time points tested and were not statistically different between time points during germination (P>0.05), with the only exception of miR159b after 48 h which was 1.5 to 2 fold higher compared to other time points (P<0.005; Fig. 4F). Together this data suggest that activity of miR159 in germinating seeds is reduced by an unknown mechanism(s) downstream of miR159 transcription and processing.

## Discussion

Plant miRNA activity is usually assumed to correlate with their abundance; that is, the higher the miRNA abundance, the stronger the miRNA activity. Here we show that this is not the case with miR159. Although its abundance in germinating seeds and rosettes is similar, miR159 acts as a “tuning miRNA” in seeds but as a “switch miRNA” in rosettes, completely abolishing target *GAMYB-like* gene expression. More specifically, our data suggests that miR159 activity is temporally attenuated during germination, suggesting the existence of a transient mechanism that modifies miR159 activity.

### *MIR159* and *MYB33* are co-transcribed in the same tissues during seed germination where miR159 acts probably as a tuner

Our results indicate that miR159 may be acting as a tuner of *GAMYB-like* expression levels during seed germination instead of as a switch. Firstly, this is indicated at the target transcript level. For instance, *MYB33* transcript levels are six fold higher in wild-type germinating seeds than in SAR and rosettes despite the levels of miR159 being similar across all these tissues (Fig. 1). Furthermore, *MYB33* transcript levels are similar in wild-type seeds and *mir159ab* vegetative tissues (Fig. 1). If a relatively constant level of *MYB33* transcription is assumed, these levels are consistent with a decrease in the silencing of *MYB33* in seeds. Another line of evidence supporting the role of miR159 as a tuner is the comparison of *GAMYB-like* transcript levels in germinating *mir159ab* and wild-type seeds (Fig. 4). *MYB33* levels were always higher in *mir159ab* but the differences compared to wild-type were only two-fold or less after 16, 24 and 32 h after imbibition, suggesting reduced silencing of *GAMYB-like* genes by miR159 at these stages of germination. In contrast, 48 h after imbibition miR159 acts as a molecular switch completely silencing *MYB33* (Fig. 2Y, Z, AA) and this is reflected in the levels of *MYB33* transcript that are six fold higher in *mir159ab* seeds compared to wild-type (Fig. 4A). A similar trend was found for the transcript levels of *MYB101* and *MYB65* during seed germination (Fig. 4B, C). Interestingly, the transcript levels of *MYB101* and the downstream target *CP1* were found to be higher in the wild-type than in *mir159ab* seeds at 16, 24 and 36 h after imbibition. This could be due to the fact that *mir159ab* seeds display a mutant phenotype [8]. They are smaller than wild-type and have a shrivelled appearance. Despite this, the germination rate of *mir159ab* seeds is similar to wild-type (Fig. 4E) and therefore the differences in gene expression that we detected are not due to a different germination rate. The effect of the absence of miR159 in germination is quite subtle, as it is the absence of *MYB33*, *MYB65* and *MYB101* [9]. This may reflect the fact that germination is highly regulated and the miR159-*GAMYB* pathway is only one component of this process.

A second line of evidence supporting the role of miR159 as a tuner in the seed is that miR159 appears to shape the MYB33 expression domain (Fig. 2). In the aleurone, the *MYB33-GUS* expression pattern during germination is reminiscent to the progression of aleurone vacuolation, a process partially controlled by this transcription factor [9]. Aleurone vacuolation starts earlier in the area of the aleurone in contact to the radicle and later on spreads to the area of the aleurone in contact with the cotyledons [12]. This is because the first event in seed germination is the radicle emergence that requires the degradation of the aleurone layer in that area. Similarly, clear *MYB33-GUS* expression is displayed in the aleurone at the radicle tip 16 and 24 h after imbibition (Fig. 2 I, O; 3 C, F). Only after 32 h when the radicle has emerged *MYB33-GUS* expression is high in the cotelydon part of the aleurone (Fig. 2 U; 3 I). This expression pattern of *MYB33-GUS* in the aleurone is not achieved just by transcriptional control, as *mMYB33-GUS* expression is very high and evenly distributed in the aleurone during seed germination (Fig. 2 A, G, M, S; 3 A, D, G). Indeed, *MIR159-GUS* expression pattern in the aleurone is reciprocal to the expression pattern of *MYB33-GUS*, being stronger in the cotelydon area of the aleurone and lower in the area in contact with the radicle at early germination stages (Fig. 2H, N, T; 3B, E, H). Therefore, our results argue that miR159 shapes the spatial and temporal expression pattern of *MYB33* in the aleurone.

*MYB33-GUS* expression was also found in the embryo 16, 24 and 32 h after imbibition (Fig. 2 L, R, X). MYB33 may be required for the elongation of embryo cells during germination, as our previous studies have shown that the up-regulation of MYB33 activates the expression of cell wall hydrolytic enzymes [9]. 5 h after imbibition no strong *MYB33-GUS* expression was seen, but *mMYB33-GUS* and *MIR159-GUS* were readily detectable in the whole embryo. This points to miR159 completely silencing MYB33. Conversely, *mMYB33-GUS*, *MIR159-GUS* and *MYB33-GUS* had similar spatial expression patterns in the embryo 16, 24 and 32 h after imbibition. However, *MIR159-GUS* and *mMYB33-GUS* lines had a stronger GUS signal than *MYB33-GUS* lines (Fig. 2 J, K, L, P, Q, R, V, W, X). This suggests that miR159 is dampening the final levels of *MYB33* in the embryo at these time points instead of shaping the spatial pattern as it appears to occur in the aleurone. Once germination is complete 48 h after imbibition, miR159 behaves as a molecular switch completely silencing *MYB33-GUS* expression in the embryo (Fig. 2 Y, Z, AA).

In summary we conclude that the silencing outcome of miR159 regulation of *GAMYB-like* expression is spatially and temporally regulated. In vegetative tissues where *GAMYB-like* function is not required, miR159 acts as a switch completely eliminating the expression of the targets. However in the germinating seed, miR159 appears to control the spatio-temporal expression pattern of *MYB33*, finely tunning its expression.

### Modulation of MYB33 expression appears independent of miR159 levels

The relationship between miR159 and its target genes is dynamic and changes in different plant organs. The question that remains to be answered is why miR159 acts as a tuner in germinating seeds in contrast to vegetative tissues where acts as a switch. One would have expected the levels of miR159 to be lower in seeds. However, we did not find gross differences in mature miR159 levels between vegetative tissues and germinating seeds (Fig. 1B, 4C). It could be possible that the levels of miR159 changes at different positions within the seed, as the pattern of *miR159-GUS* are not homogeneous. But even in the case of lower levels of the miRNA allowing more *GAMYB-like* expression to occur in the seed, this would be different to vegetative tissues were an estimated less than 10% of total miR159 is able to rescue the *mir159ab* mutant phenotype and reduce the mRNA levels of *MYB33/MYB65* to wild-type levels [7].

An alternative explanation would be the existence of an unknown mechanism regulating the activity of the miRNA without altering mature miRNA levels. To date there are few examples in which the activity of a miRNA is regulated this way. Recently a mechanism has been proposed where AGO10 sequesters miR165/miR166 in the shoot apical meristem away from AGO1, enabling expression of miR165/miR166 targets and proper shoot apical development [13]. In mice, miRNA function is suppressed in mature oocytes [14], [15]. Although mouse oocytes produce miRNAs, RNA-induced silencing complex (RISC)-loaded miRNAs and target mRNAs, miRNA-induced repression is depleted in these cells [14]–[15]. Interestingly, the siRNA pathway is unaffected in the oocytes. Although there is no an explanation for this phenomena, Suh et al. (2010) suggested that it may involve modifications to the Argonaute (Ago) proteins. It would be interesting to test if miRNA and siRNA machineries are as active in germinating seeds as in other tissues.

Another example is the differential susceptibility of the germ cell specific genes *NANOS1* and *TDRD7* to miR-430 regulation in zebrafish [16]. *NANOS1* and miR-430 are both expressed in somatic and germ cells, however *NANOS1* is more susceptible to repression in somatic cells [16]. This is due to the action in germ cells of the RNA binding protein *DEAD END 1* (*DND1*) that inhibits miR-430 dependent regulation by binding to the 3′UTR of *NANOS1* and reducing its affinity to miR-430 [17]. Another RNA binding protein, HuR has been shown to relive miR-122-induced repression of *CAT-1* by binding to its 3′ UTR upon stress conditions [18]. Similarly, a RNA binding protein may exist that reduces the ability of miR159 to bind to *GAMYB-like* mRNA in Arabidopsis germinating seeds. Alternatively, there may be factors that enhance miR159 efficiency in vegetative tissues compared to germinating seeds. Further investigations a required to determine how the different modes of action of miR159 are established.

Either way, this study clearly demonstrates that the efficacy of a miRNA can vary from tissue to tissue. This has implications for many miRNA studies. For example, transcript profiling of miRNA and mRNA populations have been used to identify miRNA target genes in many plant species, based on the assumption that mRNA abundance of target genes will inversely correlate with miRNA abundance. This study has demonstrated that this is not always necessarily the case. As very few studies such as the one reported here have analysed the abundance of miRNAs, their target transcript levels and target protein expression, how widespread this phenomena is remains to be determined.

## Materials and Methods

### Plant material and growth conditions

All *Arabidopsis thalian*a seeds were sterilized and stratified at 4°C in the dark, and then grown in 22°C growth cabinets under fluorescent illumination of 130–150 µmol·m^−2^ s^−1^ on long-day (16 h of light) or short-day (10 h of light) photoperiods in metro-mix soil. The double mutant *mir159ab* and the reporter lines *MYB33-GUS*, *mMYB33-GUS* and *miR159b-GUS* have been previously described [8], [10].

For the time course experiments Col-0 and *mir159ab* seeds were sown on 0.6% agarose plates and stratified at 4°C overnight. Then the seeds were transferred to the growth chamber and harvested at the different time points to freeze them in liquid nitrogen. For germination rate determination Col-0 and *mir159ab* seeds were sown on 0.6% agarose plates and stratified at 4°C for four days before transferring them to the growth chamber. The number of germinated seeds was scored under the dissecting microscope at different time points.

### Seed microscopy

For the histochemical localization of GUS activity seeds were sprinkled on 0.6% agarose plates and stratified at 4°C in the dark overnight, to be then transferred to a growth chamber. Seeds were harvested after 5, 16, 24, 32 and 48 h and immersed in staining buffer containing 50 mM NaPO_4_ buffer (pH 7.2), 0.2% Triton X, 2 mM Potassium Ferrocyanide and 2 mM Potassium Ferricyanide. Samples were infiltrated in this buffer under vacuum for 10 min, then the solution was replaced with new buffer containing 2 mM X-Gluc and a vacuum was applied again for 10 min. Finally, the samples were kept at 37°C overnight.

### Gene expression analysis

For determination of mRNA levels, RNA from seeds was isolated with the Plant RNA Reagent (Invitrogen) following manufacturer's instructions. RNA was precipitated overnight at −20°C with ½ volume of isopropanol and ½ volume of 0.8 M Sodium citrate and 1.2 M NaCl solution. Then the RNA was DNAse digested and cleaned with Spectrum Plant Total RNA kit (Sigma). Retrotranscriptions and quantitative RT-PCRs were performed as described [8] with primers listed in Table S1. All measurements are relative to the housekeeping gene *CYCLOPHYLIN5* (At2g29960). Mature miR159 levels were quantified with the TaqMan MicroRNA Assays (Applied Biosystems, Foster City, CA) following manufacturer instructions. For these assays, RNA from seeds was isolated as above and a 10 ng sample was retrotranscribed with TaqMan MicroRNA Reverse Transcription kit (Applied Biosystems, Foster City, CA) following the kit protocol. In each reaction, we included the stem-loop RT primers for either miR159a or miR159b and also the normalization gene *sno101*. 1.33 µl of RT-PCR product was used in 20 µl quantitative RT-PCRs. Primers sequences were all from Applied Biosystems, and are proprietary knowledge. In all quantitative RT-PCRs three technical replicates were done per sample and we analysed two different biological replicates.

## Supporting Information

Table S1 List of primers sequences used in this work.
(DOC)
