# Supplementary material for: MicroRNA159 Can Act as a Switch or Tuning MicroRNA Independently of Its Abundance in Arabidopsis
Source: PLoS One. 2012 Apr 12;7(4):e34751. doi: 10.1371/journal.pone.0034751 (PMC3325262; doi:10.1371/journal.pone.0034751)
Supplement: Table S1 — List of primers sequences used in this work. (DOC) [file pone.0034751.s001.doc]

| AGI | Gene | Forward Primer (5’-3’) | Reverse Primer (5’-3’) |
| --- | --- | --- | --- |
| At4g36880 | CP1 | ACGCTCGATAAACATTTGGG | CAAGCAAACAATCTTTCTTTGAA |
| At5g06100 | MYB33 | CCAGATAGCCATACCCCTACG | CCTCGGATTTAGTTTGGGATAC |
| At3g11440 | MYB65 | GATGATGGTTCCTGATAGCC | AGGCATCAACAGAGTCAAGG |
| At2g32460 | MYB101 | CGAGTTCTTTCCCTTTAGGACT | TGGCTCATTGTACTTGTTGTG |
| At2g29960 | CYCLOPHILIN | TGGACCAGGTGTACTTTCAATGG | CCACTGTCTGCAATTACGACTTTG |
|  |  |  |  |

**Table S1.** List of primers sequences used in this work.
